# Weak shock compaction on granular salt

**DOI:** 10.1038/s41598-024-67652-z

**Published:** 2024-07-19

**Authors:** Dawa Seo, Eric M. Heatwole, Trevor A. Feagin, Ian D. Lopez-Pulliam, Darby J. Luscher, Aaron Koskelo, Mack Kenamond, Christopher Rousculp, Christopher Ticknor, Christina Scovel, Nitin P. Daphalapurkar

**Affiliations:** 1 Theoretical Division, Los Alamos National Laboratory, P.O. Box 1663, Los Alamos, NM 87545 USA; 2 Dynamic Experiments Division, Los Alamos National Laboratory, P.O. Box 1663, Los Alamos, NM 87545 USA; 3 X Computational Physics Division, Los Alamos National Laboratory, P.O. Box 1663, Los Alamos, NM 87545 USA; 4 X Theoretical Design Division, Los Alamos National Laboratory, P.O. Box 1663, Los Alamos, NM 87545 USA

**Keywords:** Weak shock compaction, Granular materials, Force chain, Salt, Mesoscale, Mechanical engineering, Geophysics

## Abstract

This study conducted integrated experiments and computational modeling to investigate the speeds of a developing shock within granular salt and analyzed the effect of various impact velocities up to 245 m/s. Experiments were conducted on table salt utilizing a novel setup with a considerable bore length for the sample, enabling visualization of a moving shock wave. Experimental analysis using particle image velocimetry enabled the characterization of shock velocity and particle velocity histories. Mesoscale simulations further enabled advanced analysis of the shock wave’s substructure. In simulations, the shock front’s precursor was shown to have a heterogeneous nature, which is usually modeled as uniform in continuum analyses. The presence of force chains results in a spread out of the shock precursor over a greater ramp distance. With increasing impact velocity, the shock front thickness reduces, and the precursor of the shock front becomes less heterogeneous. Furthermore, mesoscale modeling suggests the formation of force chains behind the shock front, even under the conditions of weak shock. This study presents novel mesoscale simulation results on salt corroborated with data from experiments, thereby characterizing the compaction front speeds in the weak shock regime.

## Introduction

Salt (sodium chloride or halite) is an abundantly occurring mineral. The crystalline form of salt is a crucial geological material for various aspects of petroleum engineering. Salt domes formed by geological processes have commonly served as underground traps (or caverns) that hold oil and natural gas reservoirs and can act as disposal sites for hazardous waste^1–3^. The impermeability characteristics of salt make these caverns attractive sites for underground storage of hydrogen for advanced clean energy. Salt dome formation is typically facilitated by the deformation-induced flow of salt beds under tremendous pressures due to tectonic movement and the downward pressure of overlaying sediment^4,5^. Furthermore, to seal cavity shafts and to ensure the tightness of the salt repository, compacted granular salt can be applied as a backfill material^2,6,7^. With the advances in clean energy, understanding the mechanics of compacting granular salt is increasingly crucial for repository design and safety assessment against hazards, such as earthquakes and impact loads. Furthermore, shock compaction of granular materials is of great interest in dynamic processing fields such as powder metallurgy^8^, geophysical flows^9,10^, and ballistics^11,12^. In context, this study focuses on characterizing granular salt’s weak shock compaction response.

A continuum approach for modeling a granular material considers material and void volumes as a single-component system^13^. It assigns averaged properties by ignoring the characteristic processes and features underlying the heterogeneous nature of deformation, such as inelastic particle-scale interactions and morphological changes associated with the void collapse. We will use the term ‘grain’ to refer to a domain of a deformable solid material when the focus is on sub-grain deformation and other intrinsic characteristics and the term ‘particle’ to refer to the grain when the focus is on averaged property or interactions extrinsic to its surroundings. In the continuum approach, additional constitutive relations, such as the P-$\alpha$ model and its variations induced from the relationship between pressure and density, are typically used to describe compaction^14^. While such a continuum model has been useful, its averaging scheme ignores the heterogeneous nature of grain-scale processes that can significantly affect the dynamic bulk response^15^. Secondly, for a material with a distribution of defects, the tail end of the distribution, rather than the mean, often has a dominant effect on the variation of a continuum property^16^. It is therefore essential to explore the grain-scale responses and their effect on the bulk response, to fully understand the mechanics of granular systems. Mesoscale modeling simulates individual particles explicitly, enabling sub-grain analysis and accounting for the physically based inelastic deformation behavior of the grains undergoing large deformation.

Prior efforts on computational modeling to resolve varied aspects of the grains used techniques such as hydrocodes^17,18^, finite element method (FEM)^19^, discrete element method (DEM)^20^ and Material Point Method^21^. For example, Borg and Vogler (2008)^22^ explored transmission of the compaction wave through granular tungsten using two-dimensional numerical simulations with an Eulerian mesh. Eulerian methods use fixed discretized cells, and each cell can contain multiple and dissimilar materials. This feature allows large deformation of multi-material interfaces under shock compaction. However, the Eulerian method is limited in simulating realistic grain-on-grain contact dynamics. Borg and Vogler (2008)^22^ noted the importance of considering spatially and temporally averaged quantities for the continuum description of granular materials. Furthermore, they addressed the reason behind a much slower compaction wave in a porous material than the speed of shock in a pristine material attributed to the transport mechanism that closes the gaps and carries the compaction process forward by grains. A previous study by Borg and Vogler (2009)^23^ conducted a parametric study to explore the variation and sensitivity of the computationally derived dynamic response characteristics to micro-scale material properties. The results showed that bulk response is sensitive to pore volume fraction, the pristine material’s dynamic yield strength, and particle arrangement.

We further note the importance of grain morphology on the response of shock-compacted granular materials. Perry et al. (2016)^24^ examined the effects of different morphologies and concluded that the morphological properties dominated at low strain rates, stiffening the material’s response with increasing roughness. Derrick (2018)^25^ conducted a mesoscale simulation with different grain shapes. The author found that the angle of the grain face to the shock front is directly related to the temperatures and pressures experienced on its shock-facing side. The study by Fredenburg^26^ on various fine powder morphologies indicated that high-strain rate loading resulted in a compaction response that was similar for all morphologies; however, for low stress and low strain rates, particle morphology had an influence on the compressive response of powders. Such findings from mesoscale modeling have expanded our understanding; however, the weak shock compaction in granular materials is one such regime that remains less understood because of the comparable effects of both strength and hydrodynamics.

**Figure 1:**
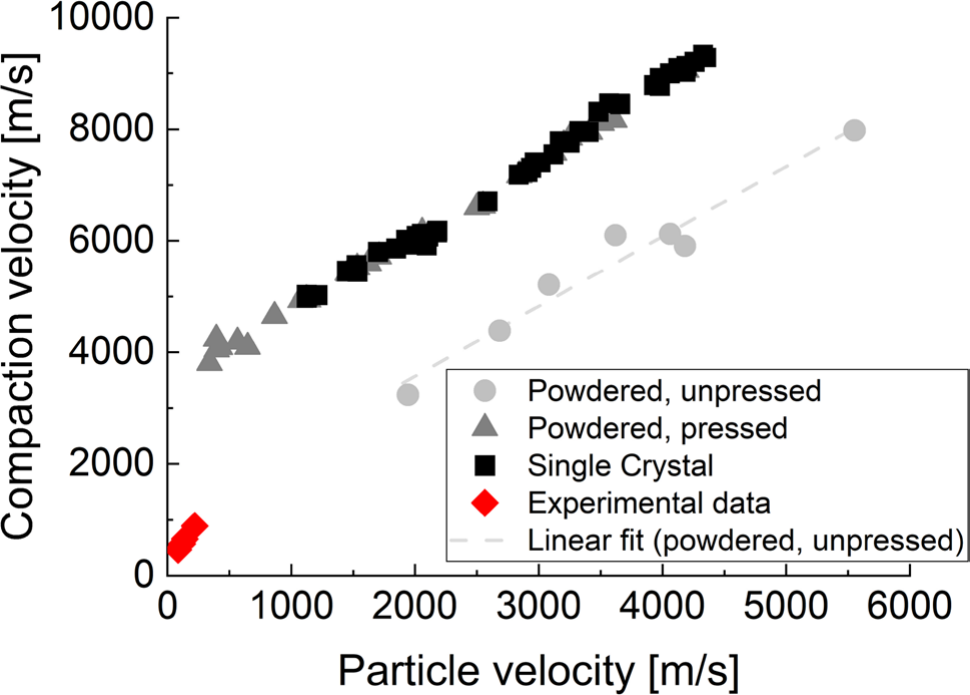
Shock-compacted data on various forms of granular salt after Marsh (1980). Superimposed is the experimental data on weak shock compaction of salt from this study (marked in red).

The shock wave front separates the compressed material from the material that is not compressed. The traveling velocity of the shock wave front within the material is referred to as the shock velocity ($U_s$). The motion of a material particle in the compressed, post-shock regime is characterized using a particle velocity ($U_p$)^27^. Figure 1 shows a plot with experimental data on measured $U_s$ against $U_p$ obtained for unpressed salt (for a range of initial densities $\rho _0=0.61-0.99 \text { g/cm}^3$), pressed salt (average initial density $\rho _0=2.137 \text { g/cm}^3$), and a salt crystal (average initial density $\rho _0= 2.163 \text { g/cm}^3$)^28^. Note that Marsh’s paper^28^ does not differentiate between the tap densities for linearly fitting a $U_s$-$U_p$. There is significant uncertainty in the form of the $U_s$-$U_p$ for granular materials, especially in the weak shock regime. For example, Marsh’s (1980)^28^ data on salt demonstrates a need for a quadratic form of the $U_s$-$U_p$ for a pressed granular salt specimen^29^. We interpret that all the voids in the pressed specimen have been eliminated because the results match the single crystal NaCl. A recent study on computational modeling suggested that the slope of $U_s$-$U_p$ would change in the weak shock regime for granular tungsten carbide (WC); thus, the $U_s$-$U_p$ curve for an unpressed granular material is expected to be nonlinear^23^. In this regard, there is a need for a fundamental understanding of the response in the weak shock regime for granular materials. Moreover, in this study, we have not constrained the testing to the range of tap density used by Marsh^28^ because of the lack of available data on the types and grain size distribution of salt, which is ultimately important for making direct corroborations. Therefore, this paper aims to provide shock-compacted granular salt data in the weak shock regime rather than extending Marsh’s data.

The present study offers important weak shock compaction experimental data and mesoscale insights from simulations on granular salt. We conducted a linear shock compaction test to examine salt’s response under the low impact velocity range. The linear compaction setup consists of an elongated bore length, facilitating the visualization of a propagating shock through the sample that further enabled characterization of the evolution of the shock compaction response in a granular system. Figure 1 shows the experimental data attributed to this work, indicated in red markers. Mesoscale simulations were used to examine the evolution of grain-scale response, such as the development of a precursor, formation of force chain (or stress bridging) in the precursor and behind the shock front, shock wave substructure velocities, leading front thickness, and aspects of heterogeneity within the shocked granular system, which were otherwise unavailable from the test data. The details of experimental methods and computational simulations are described in the methods section, following the section on conclusions.

## Results

### Experiments

**Figure 2:**
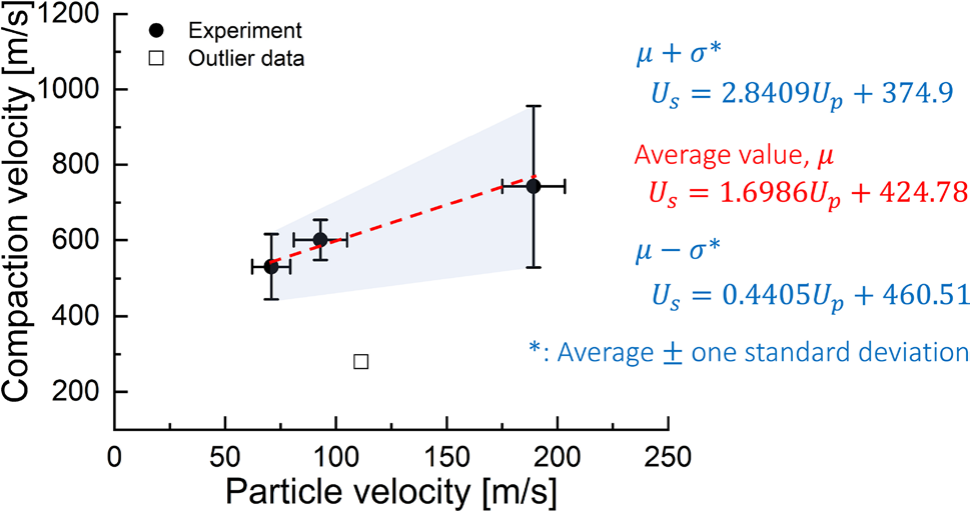
Shock compaction versus particle velocities analyzed from experimental data on table salt.

Shock compaction velocities and corresponding average particle velocities were measured for individual impact tests. Figure 2 presents the averaged values of shock compaction velocity $U_s$ and particle velocity $U_p$ for all impact velocities and their variation representing one standard deviation; the details of calculations are included in the supplementary material. The variation results from the modulated compaction and particle velocities as the shock compaction wave experiences varying spatial distribution of particles as it traverses over the specimen length.

The linear fit to the average values was obtained after excluding the outlier data from the impact velocity of 155 m/s. The linear fit yielded a $U_s = 1.6986 U_p + 424.78$ m/s. The outlier may be due to an unexpected variation in the sample packing; hence, we did not consider this test result in our data analysis. Our linear fit accounting for variability can be represented using lower and upper bounds as $U_{s-low} = 0.4405 U_p + 460.51$ m/s and $U_{s-high} = 2.8409 U_p + 374.9$ m/s, respectively. Marsh found $U_s = 1.2542 U_p + 1064.6$ m/s, which has a slightly lower slope than our experimental result in the weak shock regime.

**Figure 3:**
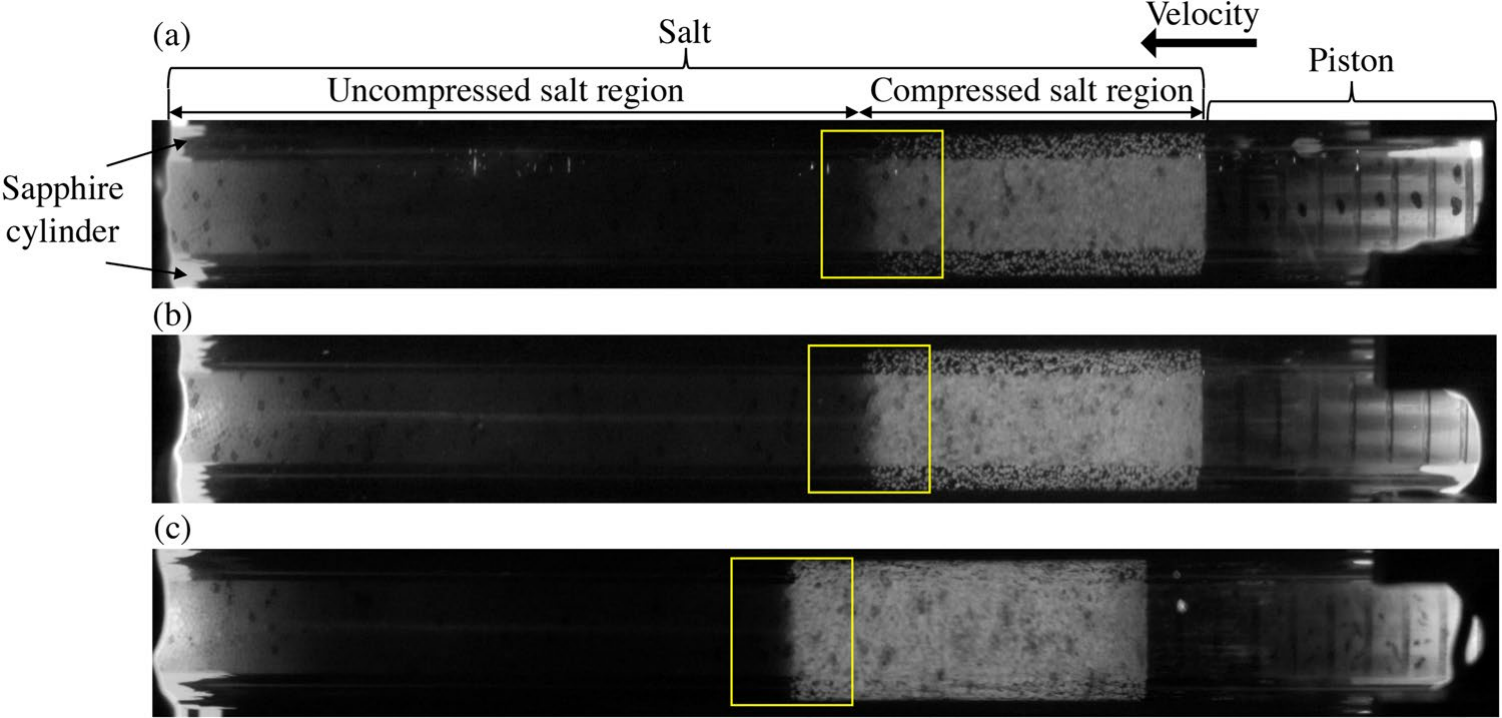
High-speed images from experiments showing moving shock compaction wave at a time of 53.4 µs from impact, for impact velocities of (**a**) 95 m/s (**b**) 125 m/s, and (**c**) 245 m/s. Note the difference in the appearance of the front shape. We find a possible explanation in the simulations.

The shock compaction responses for three impact velocities were compared from high-speed imaging at 53.4 μs from the time of impact, as shown in Fig. 3. The location of the shock compaction leading edge is indicated by a yellow box. Figure 3c shows a uniform shock-compacted regime with a sharp leading edge exhibited by a steep light gradient for the case with the impact velocity of 245 m/s, in contrast to a more diffuse leading edge that has a shallow light gradient for the case with the impact velocity of 95 m/s in Fig. 3a. This sharpness in the shock front for the higher impact velocity indicates the relatively short distance over which the particle velocity ramps up to the highest value that occurs behind the shock compaction wave. For the case with an impact velocity of 125 m/s, as shown in Fig. 3b, the leading edge of the shock compaction wave was an intermediate case, i.e. not as sharp as the case with a higher impact velocity of 245 m/s, but the gradient is still somewhat stronger compared to the case with the lower impact velocity of 95 m/s. We infer from these results that the case with the lowest impact velocity requires a greater distance (or a longer time) to evolve a shock compacted state compared to those with other cases with higher impact velocities.

### Mesoscale modeling

We developed a mesoscale computational model to simulate the shock compaction test with square-shaped grains using the FLAG^30,31^ hydrodynamic code. The Lagrangian method was preferred because interface tracking is natural to this method, and physically based contact models can be employed. Since impact velocities were in a weak shock regime, we did not anticipate any issues related to mesh distortion. The simulations employed a statistically representative size distribution for square-shaped particles within a two-dimensional simulation setting. Frictional forces, both the friction between grains and the friction between the salt and tube walls, were not accounted for in the model and might be responsible for some differences between the experiment and modeling. Results from the hydrocode were used to analyze the grain-scale response of salt under low-impact velocities. We developed an averaging technique for state variables suitable for the continuum description. Simulation results on shock compaction velocities were validated against the test data.

**Figure 4:**
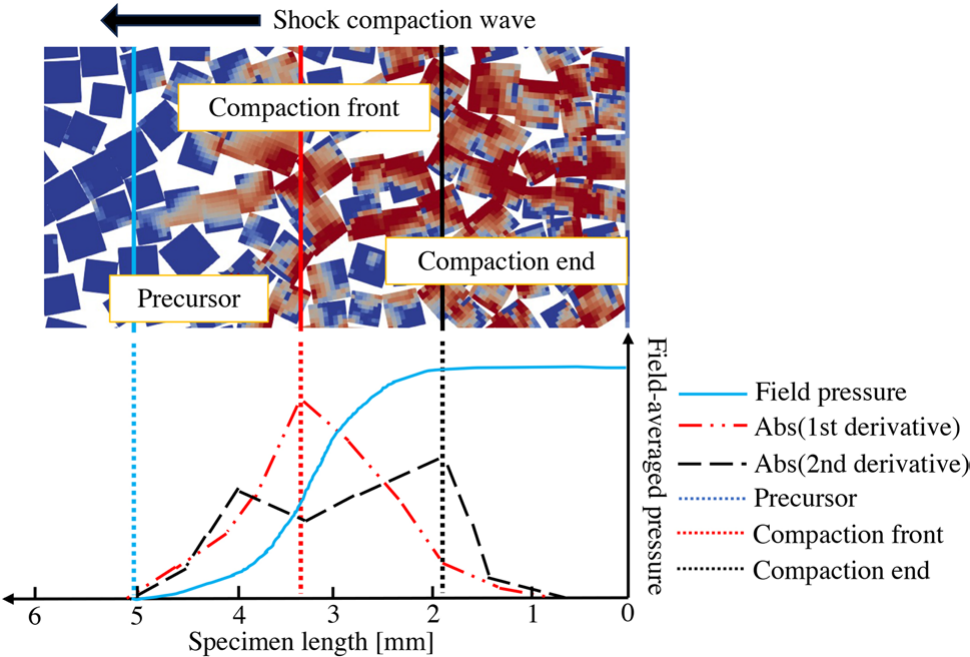
(Top) Enlarged view of zone pressure distribution for an impact velocity of 95 m/s and (Bottom) averaged (field) pressure magnitude, and its first and second derivatives across the leading edge of the shock wave to identify locations of the precursor, compaction front and end.

Figure 4 (Top) displays a representative result of zone pressure distribution for an impact velocity of 95 m/s. Significant spatial variation in zone pressure appeared within the propagating shock compaction wave. Figure 4 (bottom) is a plot of averaged pressure and identification of three substructures of the shock compaction wave; as is common to a shock wave, the pressure gradually increases from zero pressure in front of the shock wave to the shock compaction pressure behind the shock through a gradient that we will refer to as the ramp in pressure.

The pressure ramp within the shock wave can be identified using three substructures: precursor (Eq. 4), compaction front (Eq. 5), and compaction end (Eq. 6). The precursor indicates the location of a leading edge, which can be identified by a finite pressure front. The front is due to the propagating elastic wave, which builds pressure in its wake. A noteworthy aspect of this process is the pressure built up by forming force chains, also called stress bridging^32^. Force chain phenomenon has been observed in granular systems, but observations were typically limited to quasistatic conditions^22,33–35^. In this regime, particle rearrangement occurred through displacement and rotation, resulting in particle networks that transmitted stress, while the deformation was largely elastic with a minor amount of plastic deformation. Next, the compaction front was defined as the transition edge where most particles undergo intense pressure change due to confined loading along the specimen length; this can be considered representative of the actual shock compaction wave. Finally, the shock compaction end was identified by shock-compacted particles reaching pressure that stays nearly consistent behind the shock.

**Figure 5:**
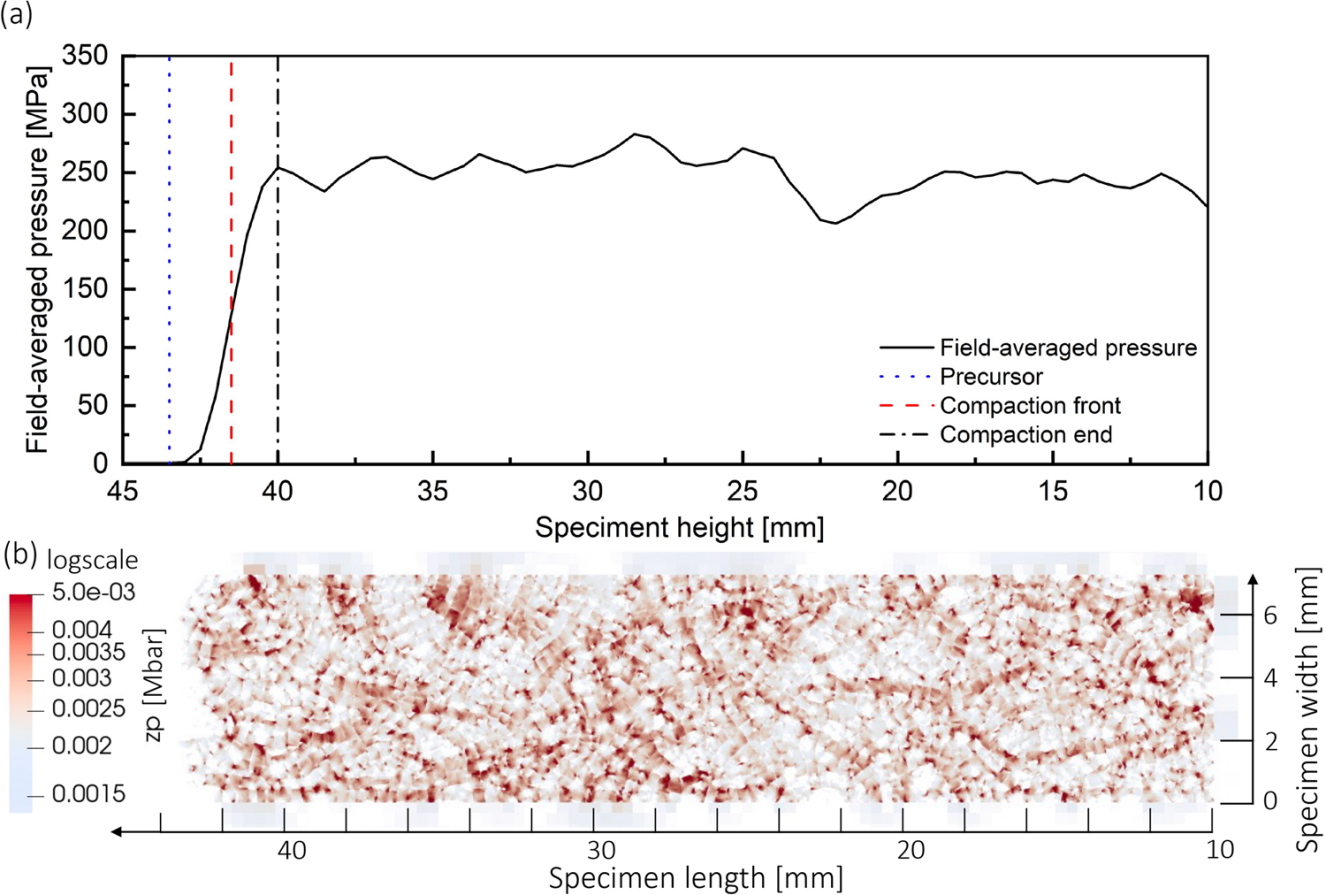
(**a**) Evolution of 1D averaged (field) pressure along the specimen length, along with the position of the substructures precursor, compaction front and end; (**b**) visualized zone pressure (before averaging) at 47 μs under an impact velocity of 245 m/s showing force chains in the shock compacted region.

Figure 5a shows a profile of the averaged field pressure along the specimen length at 47 μs under an impact velocity of 245 m/s. Figure 5b shows a corresponding snapshot of the zone pressure (raw data before averaging), $z_p$, defined as pressure corresponding to a finite volume element within a material domain. The substructure of the shock compaction wave identified using locations of the precursor, compaction front, and end are indicated as vertical lines. Specifically, the precursor given by Eq. 4 accurately indicated the position of the head of the force chain (see the "Methods" section). Behind the precursor, major force chains begin to develop before the compaction front. As one moves away from the precursor (along the *x*-axis), several more force chains developed by the passing compaction front during the ramp phase of the compaction wave, implying increased pressure being communicated to the neighboring particles, the communication speed being a fraction of the pristine material wave speed in the granular medium^36^. With increased pressure, newer regions come in contact and act as pathways for stress bridging. Table 1 summarizes the simulation results with average measures for the shock compaction response and one standard deviation. The *average field pressure* was calculated by spatial averaging of the pressure field estimated in the post-compaction regime, i.e., behind the shock compaction front.

**Table 1:** Summary of experimental and mesoscale simulation results.

Measured from the experiment				
Impact velocity	95 m/s	125 m/s	155 m/s	245 m/s
Particle velocity, $U_{p}$ (m/s)	70.80 ± 8.58	93.01 ± 12.04	-	189.20 ± 14.10
Shock velocity, $U_{s}$ (m/s)	530.59 ± 85.47	600.54 ± 53.04	-	742.82 ± 213.70
Estimated from the simulation				
Post compaction density, $\rho _s \text { (g/cm}^3)$	1.85 ± 0.085	1.90 ± 0.082	1.94 ± 0.084	2.02 ± 0.068
Average field pressure, $\rho _{avg}$ (MPa)	51.42 ± 2.93	77.49 ± 16.46	113.81 ± 17.88	242.04 ± 30.45

**Figure 6:**
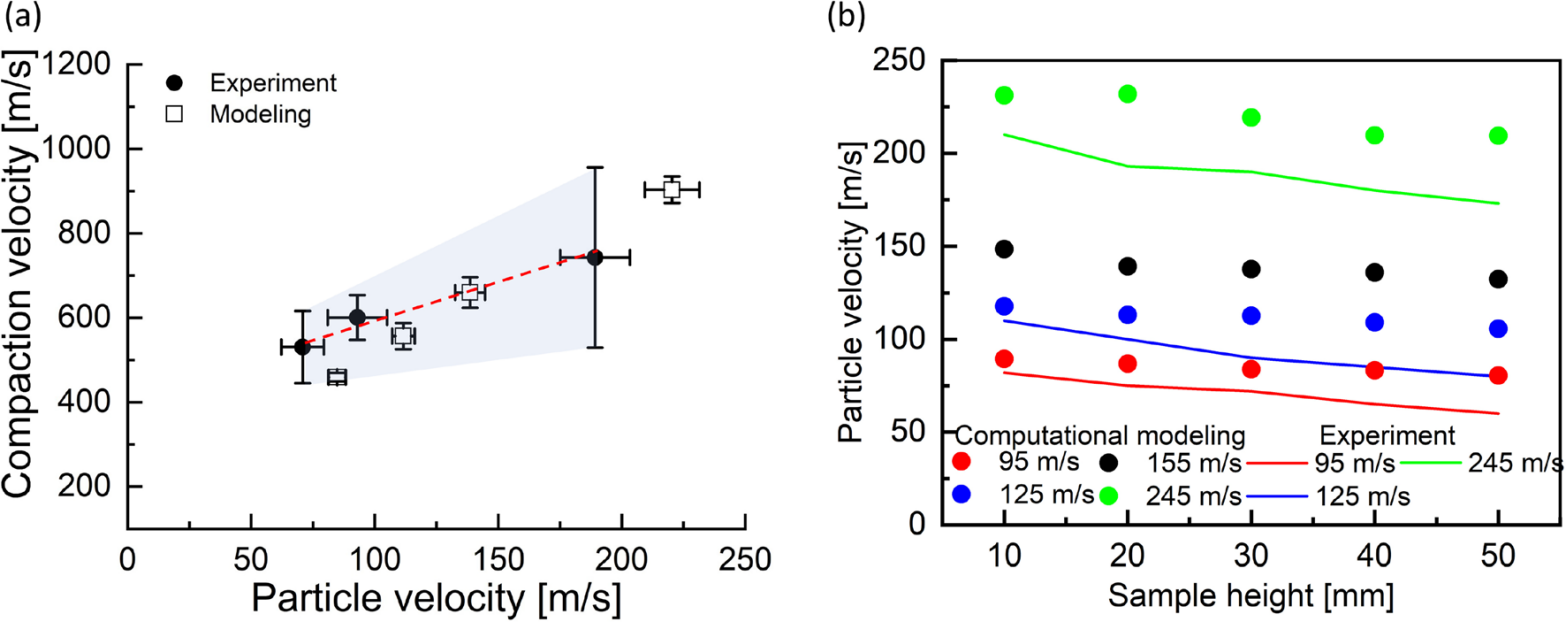
Comparison between experiment and simulated results (**a**) shock compaction and particle velocities, $U_s-U_p$ (**b**) evolution of particle velocity with distance traveled by the shock wave for different impact velocities. Simulated results agree well with experimental data in (**a**). The particle velocities with shock wave travel distance have a reasonable comparison with experiments in (**b**).

Figure 6a compares compaction and particle velocities of computational modeling with experimental data for four cases of impact velocity. This study achieved a reasonable quantitative comparison of $U_s-U_p$ responses between computational modeling and experimental data, as shown in Fig. 6a. The $U_s-U_p$ slope from modeling was stiff compared to the experimental data, which may be attributed to several sources. This discrepancy in slope might be induced by uncertainty in the yield strength^23^ assigned to the single salt grain and lack of a fracture model^37^ in the mesoscale simulation. The porosity relation between experiments (3D) and computational modeling (2D) may not be sufficiently captured using the stereology analysis, Eq. 2.

Figure 6b profiles the evolution of particle velocity along the specimen length over a 10 mm interval along the *x*-axis. Both experiment and simulation results present a trend of decreasing particle velocities as the shock compaction wave travels down the specimen length. Data from simulations is again in reasonable agreement with experimental measurements for all cases of impact velocities, although simulations consistently over-predicted the particle velocity, perhaps indicating the importance of frictional dissipation.

**Figure 7:**
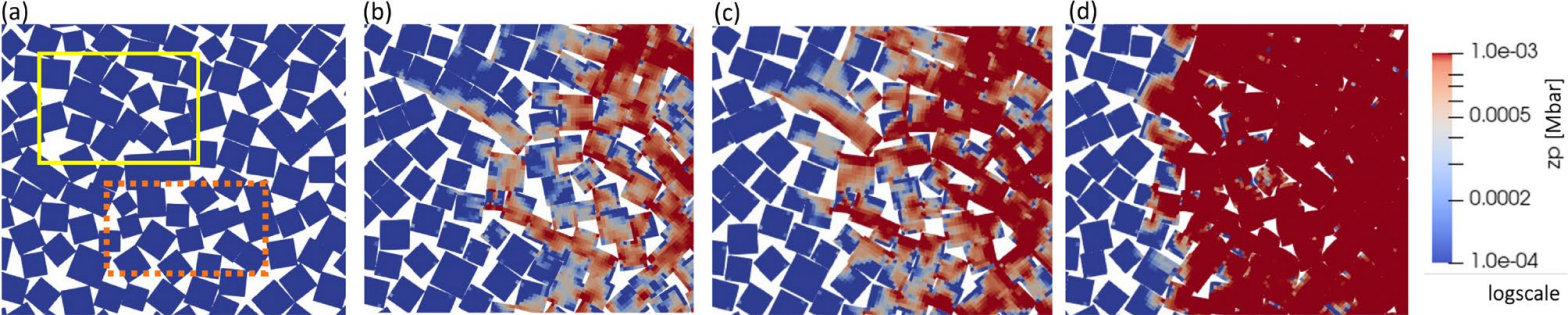
Enlarged view of the particles undergoing shock compaction, located at $\approx$ 30 mm from the piston, for (**a**) Initial configuration, and impact velocities of (**b**) 95 m/s (**c**) 125 m/s and (**d**) 245 m/s. Snapshots (**b**–**d**) show the formation of force chains in the precursor. The precursor is relatively more heterogeneous at lower velocities in (**b**) and (**c**) compared to (**d**).

The effect of impact velocity on the propagating shock in the granular salt is shown in Fig. 7. Figure 7b, c, and d are snapshots from simulations at 65 μs, 55 μs, and 33 μs, showing particles colored using zone pressure under shock propagation for impact velocities of 95 m/s, 125 m/s and 245 m/s, respectively. We made the following observations about communication between particles that had close face-to-face contact between particles in the initial configuration; a set of particles is indicated with the yellow box in Fig. 7a. For the lowest impact velocity of 95 m/s, face-to-face contact was the preferred particle arrangement for developing a force chain over other types of contact, such as point-to-face or point-to-point contact, as shown in Fig. 7b. This observation implies that the contacting condition resulting from initial particle arrangement and relative orientation determines the preference for force chain formation in granular materials, especially in the weak shock’s precursor stage. We observed yet another case; the face-to-face contact between particles was acquired through rearranging particles initially in the corner-to-face contact condition, as shown in Fig. 7a, indicated using the orange box bounded by the dashed line. Interestingly, even these particles contributed to forming a force chain, as shown in Fig. 7c.

Despite the same initial condition of particle arrangement as in Fig. 7a, a higher impact velocity of 245 m/s resulted in a more uniform precursor due to a relatively higher number of force chains, as shown in Fig. 7d. Specifically, relatively uniform pressure distribution under an impact velocity of 245 m/s contrasts with the substantial pressure heterogeneity in the shock wave’s precursor, where only a few force chains appear at 95 m/s (Fig. 7b), with moderate force chains appearing at 125 m/s (Fig. 7c). The density of force chains, which may be defined as the total length of force chains per unit area, was influenced by varying impact velocities. Higher impact velocity leads to a higher density of the force chain, giving a uniform appearance of the pressure in the precursor. Based on Daniels’ study^38^, interparticle contact areas along force chains provide the main conduit for the propagation of an acoustic pulse. From a continuum perspective^39^, the velocity of the propagating compaction wave can be thought to depend on the driving force due to the jump in the pressure across the moving compaction wave. The decreased density (homogeneity) of force chains can be interpreted as a reduction in the pressure (behind the compaction wave), which consequently correlates with lower compaction velocities. This phenomenon is analogous to the experimental observation in Fig. 3, having a uniform intensity in the shock-compacted region with a stronger contrast gradient at the shock’s leading edge that appeared in the case of higher impact velocity (see Fig. 3c).

We note the following feature related to the evolution of force chains due to the relative motion between particles. Flat surface features (associated with the square shape) limit particle mobility in rotational degrees of freedom once they form face-to-face contact with other particles. This results in a cluster of particles behaving like a lumped solid. Interestingly, it was found that those particles that acquired face-to-face contact in the initial configuration contributed to the heterogeneous nature of the shock front due to the generation of the force chains that separate and move ahead of the primary shock front. These observations are consistent with the effect of porosity on the 1D wave speed in a chain of particles^36^; however, face-to-face contact between square particles precludes the effect of porosity.

**Figure 8:**
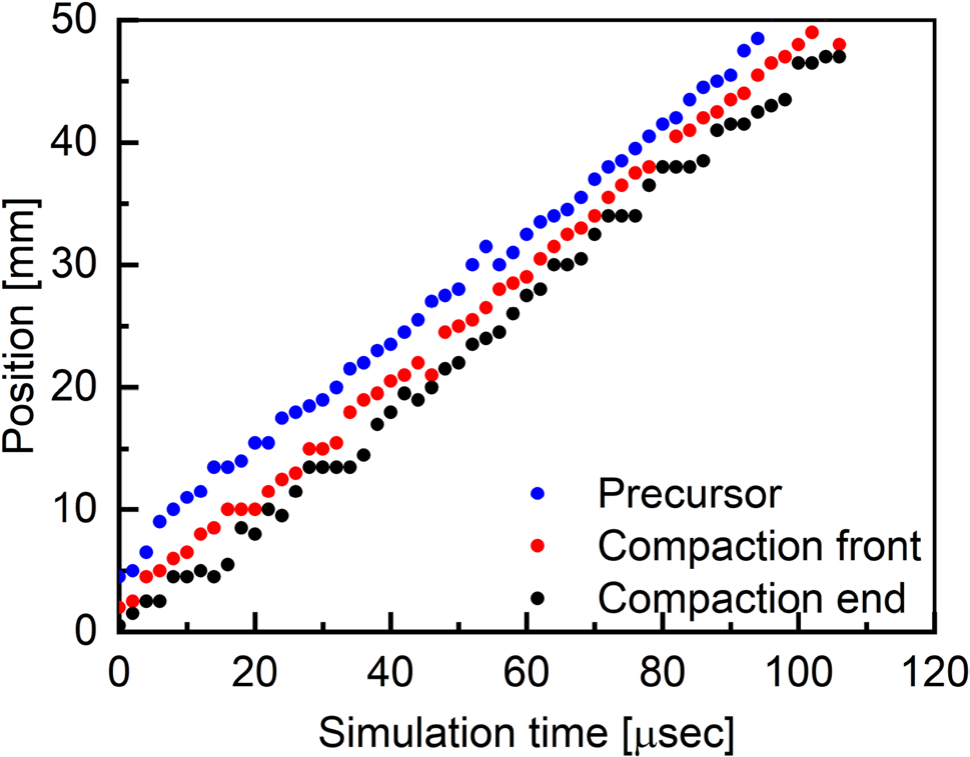
Evolution of shock compaction wave indicated using position history from simulations with an impact velocity of 95 m/s. Evolution is shown for three shock substructures: precursor (blue), compaction front (red), and compaction end (black).

Figure 8 shows the position history for the three shock substructures. This result is plotted for the impact velocity of 95 m/s as a representative since other impact velocities exhibited similar trends. Position evolution is shown in Fig. 8 for all three shock substructures. The slope of their corresponding straight line would indicate the instantaneous propagation velocity of these substructures. Once the substructures of the shock are formed (around 5 $\mu$s), their instantaneous velocities remain the same over significant distances, implying the compaction wave has reached a steady state motion.

**Figure 9:**
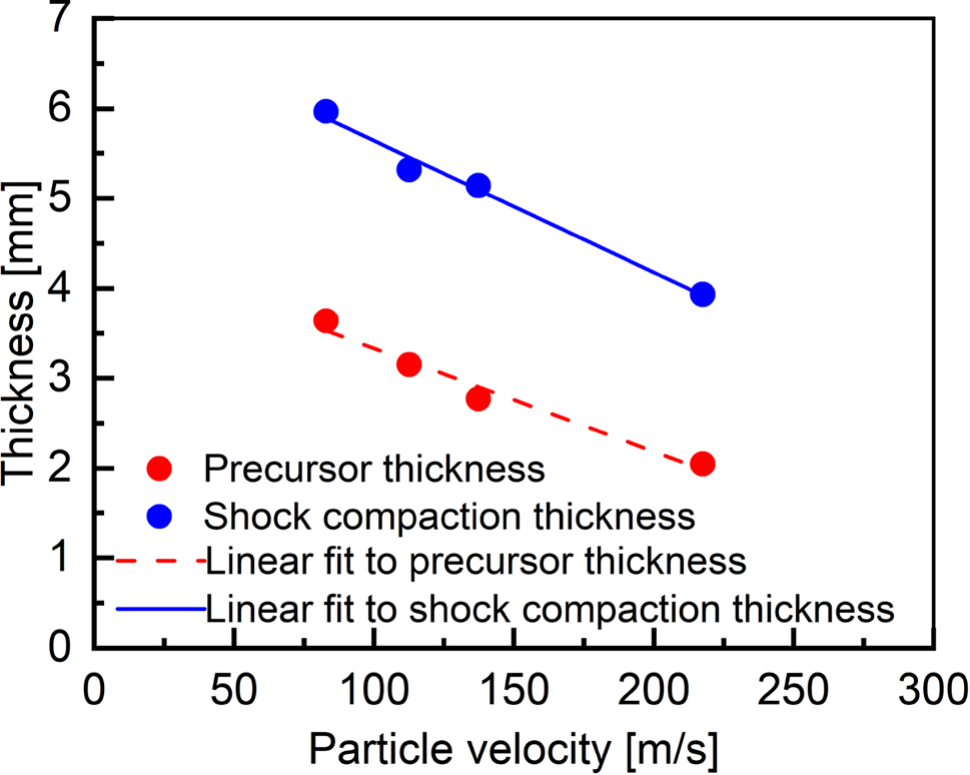
Effect of impact velocity on simulated precursor thickness (red) and shock compaction thickness (blue). Thicknesses reduce with increasing impact velocity. See Fig. 4 for the image of the wave structure.

To investigate the length of shock development in granular samples at different impact velocities, two types of thickness were calculated: precursor thickness and shock compaction thickness (as shown in Fig. 9). Precursor thickness was measured from the precursor to the compaction front, signifying a region where the developing force chain is dominant. Shock compaction thickness was calculated as the distance from the precursor to the compaction end, signifying the representative length of the shock compaction wave over which pressure ramps from the initial unshocked condition to reach the shocked condition. From modeling and experimental observations (as shown in Figs. 7b-d, and 3), it was found that low impact velocity displays broader shock compaction thickness associated with the high heterogeneity of pressure distribution. For the impact velocity of 245 m/s, the reduction in precursor thickness is 44% and the reduction of shock compaction thickness is 34% compared to corresponding thicknesses at an impact velocity of 95 m/s.

**Figure 10:**
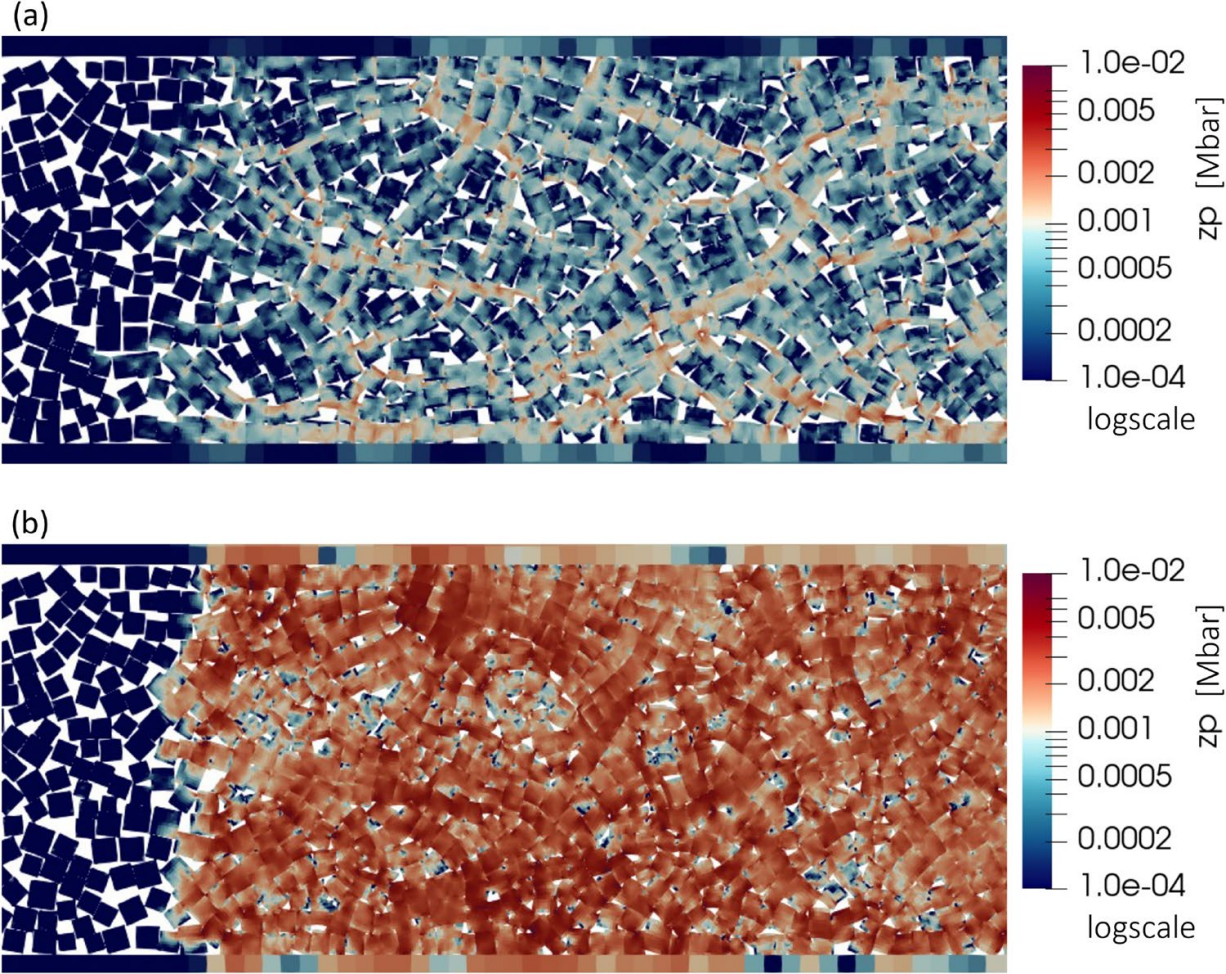
Comparison of shock compacted regions indicating the formation of force chains between impact velocities of (**a**) 95 m/s and (**b**) 245 m/s. Higher impact velocity leads to a higher density of force chains and a higher magnitude of pressure.

The pressure distribution in granular salt remained uneven, and the force chain network persisted strongly in the post-shock compaction regime. In the case of simulations utilizing spherical grains^22,32^, the shock propagated through the shortest available path via contacting particles, leaving some portions of the grains experiencing lower pressure, resulting in significant spatial variations. In the present study, square-shaped particles exhibited intense pressure within the force chain with predominantly face-to-face contact. A highly heterogeneous pressure distribution was observed at an impact velocity of 95 m/s, as seen in Fig. 10a. At an impact velocity of 245 m/s, most particles were involved in the particle network forming force chains, and the pressure distribution was significantly uniform, as seen in Fig. 10b. For the lower impact velocity, a significant portion of force chains were oriented along the direction of compression, i.e. the *x*-axis, as seen in Fig. 10a. This observation was consistent with the data on granular spheres obtained by Bardenhagen and Brackbill (1998)^35^. Complete densification was not achieved in the post-shock compaction regime, indicating that an impact velocity above 245 m/s is necessary to remove the voids completely. Our results are consistent with other studies, e.g., granular tungsten carbide required a 450 m/s driver plate to remove all voids in the study by Borg and Vogler (2008)^22^.

## Discussion

This study provided insights into the mesoscale response of square-shaped grains by exploring bulk and particle-scale responses under weak shock compaction. From simulations, complete densification was not achieved for granular salt under weak shock compaction. In the compacted regime, strong force chains persisted through contacting grains while some particles were left to transmit weak pressure, resulting in heterogeneity of pressure distribution in the particle network. As impact velocity increased, more particles got involved in developing force chains. In future studies, it may be instructive to examine higher impact velocities to collapse voids and investigate the mechanics of spatial distribution of shear stress in cube-shaped grains.

High-speed imaging of experiments facilitated the examination of the effect of impact velocity on the uniformity of the evolving shock compaction front. A robust contrast regime was identified between the pre- and the post-shock compaction at an impact velocity of 245 m/s. On the other hand, a compaction regime with a lower contrast gradient was observed at an impact velocity of 95 m/s. These relative differences in contrast gradient indicate that a relatively larger specimen length is required for a granular material to achieve a steady shock condition with decreasing impact velocity. The longer specimen length in our setup enabled the characterization of time-dependent particle velocity over a greater distance of shock wave travel, which is otherwise unavailable in the flyer-plate setup.

Although the simulation predicted shock compaction velocity and particle velocity matched reasonably well with the experimental data, we did not conduct optimization of material strength or friction parameters for the model to fit into the experimental data. In the linear compaction experiment, particle velocity decreased with the distance traveled by the wave, contributing to variability in the characterized $U_s-U_p$ relation. Mesoscale simulations predicted $U_s-U_p$ in agreement with the experiments, notwithstanding a stiffer slope. Future studies may consider friction and fracture to model a more accurate response of granular salt under shock compaction. Such optimized mesoscale modeling will be valuable in predicting the precise shock-compacted response of granular salt without available data, especially for weak shock.

## Conclusions

This research investigated the weak shock response of granular salt using both experimental and mesoscale simulation approaches. Mesoscale simulations were compared to the shock experiments, which characterized the velocity of the shock compaction front using a linear compaction setup. Particle image velocimetry was used to analyze data from the high-speed images, and the resulting characterized the $U_s-U_p$ relation as $U_s=1.6986 U_p + 424.78$ m/s. The simulation and experiment results showed consistent trends in the $U_s-U_p$ behavior of the salt, and the data from the experiments validated the simulated results. As impact velocity increased, the shock compaction front thickness decreased. This finding aligned well with the experimental observation about the decreasing span of the contrast gradient with increasing impact velocity regarding the shocked region’s leading front.

A pressure field was used to identify three substructures of a shock compaction front, namely precursor, compaction front, and compaction end, further enabling the identification of precursor thickness and shock compaction thickness. Both the thicknesses decreased with increasing impact velocity.

Force chains were identified as the load transfer mechanism in the shocked region. Further, force chains that led to heterogeneity in the shock compaction front were identified in the precursor, especially at low impact velocity (95 m/s). Most force chains were developed through face-to-face contact, while higher impact velocities resulted in particle deformation that resulted in evolving force chains. Force chain evolution was thus amplified by increasing impact velocity.

## Methods

### Linear shock compaction test on salt

**Figure 11:**
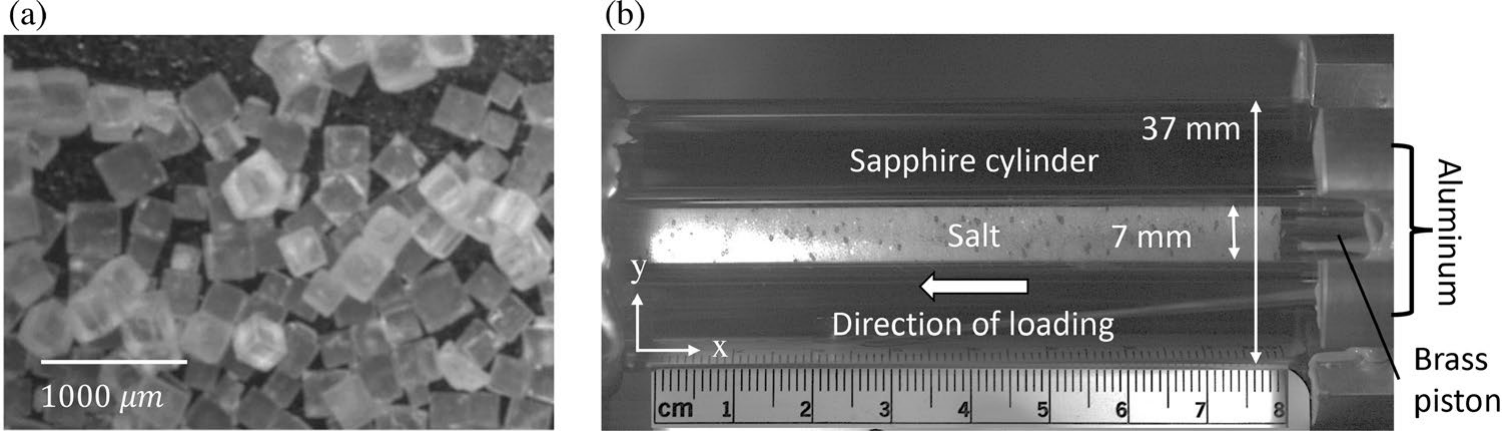
(**a**) Optical image of the granular salt used for testing (**b**) Experimental setup of linear compaction test - light source from left floods inside the sapphire. Brass piston, upon impact, travels negative x-axis.

Shock compaction tests were conducted on Morton’s non-iodized table salt (estimated water content < 0.1 %) having a density of single grain, 2.165 g/c$\text {m}^3$ sieved between 0.25 mm and 0.5 mm (average length $L_{avg}= 0.375$ mm). An optical image of the salt grains used in this study is presented in Fig. 11a. The shock test utilized a hollow sapphire cylinder (density of 3.98 g/c$\text {m}^3$) with an inner diameter of 7.0 mm, an outer diameter of 37.0 mm, and a length of 80.0 mm. This elongated bore length facilitated visualizing propagating shock over a considerable distance in the sample. A smaller bore diameter enabled experiments on a manageable number of grains that could be explicitly simulated. Note that we expect an edge release to occur within several mm of the sample length. Unlike the edge release in a monolithic specimen that occurs only at the specimen edges, the edge release in porous granular material can occur at the edges of the grains throughout the specimen, resulting in a heterogeneous stress and strain distribution. The deviation from the one-dimensional strain condition may reduce with the decreasing porosity of the specimen, which is likely only reasonable sometime after loading begins for highly compressed granular material. Consequently, the integrated simulation and experiment presented in this work are of great utility in characterizing the heterogeneous nature of the response that is expected to occur. The cylinder provided sufficient confinement, thereby preventing the transverse expansion of the sample under shock. A sample preparation technique called dry pluviation was adopted to prepare an initial packing by *raining* (pouring) grains under gravity^40^. To ensure uniform salt densification, the appropriate salt mass was dry pluviated and lightly compressed into the sapphire tube to form 65% relative density (resulting in the initial density of granular salt as $\rho _0= 1.407 \text { g/cm}^3$), 7 mm long aliquots. The prepared cylindrical sample of salt was placed into an aluminum fixture (density of 2.7 g/cm^3^), which joins the muzzle of the powder gun with the bore of the sapphire cylinder. The sample is kept co-axial with the bore of the powder gun, allowing the accurate introduction of the impacting piston made of brass (density of 8.47 g/cm^3^) into the salt column. The location of the interface between the brass piston and salt column is shown in Fig. 11b. Four different impact velocities were applied using a powder gun, resulting in velocities of the brass piston measuring 95, 125, 155, and 245 m/s (piston indicated on the right side in Fig. 11b) as it traveled to the open end of the cylinder towards the left in Fig. 11b. High-speed imaging was used to record optical images to identify the moving shock front’s position, which was subsequently used to calculate the velocity history of a shock compaction wave. High-speed images were also used to analyze particle velocities using the particle image velocimetry (PIV) technique. To facilitate data analysis using PIV, several salt grains were colored black, as seen in Fig. 11b. The response of salt under shock compaction was imaged at every 2.67 µs interval. The light source on the left in Fig. 11b illuminated the sapphire tube. The flood light in the sapphire tube reflects off the grains that push up against the lateral wall. The full densification of the salt region in the wake of the shock compaction wave results in higher illumination of the grains in the vicinity of the lateral wall compared to their unshocked state.

### Experimental data analysis: compaction and particle velocity

**Figure 12:**
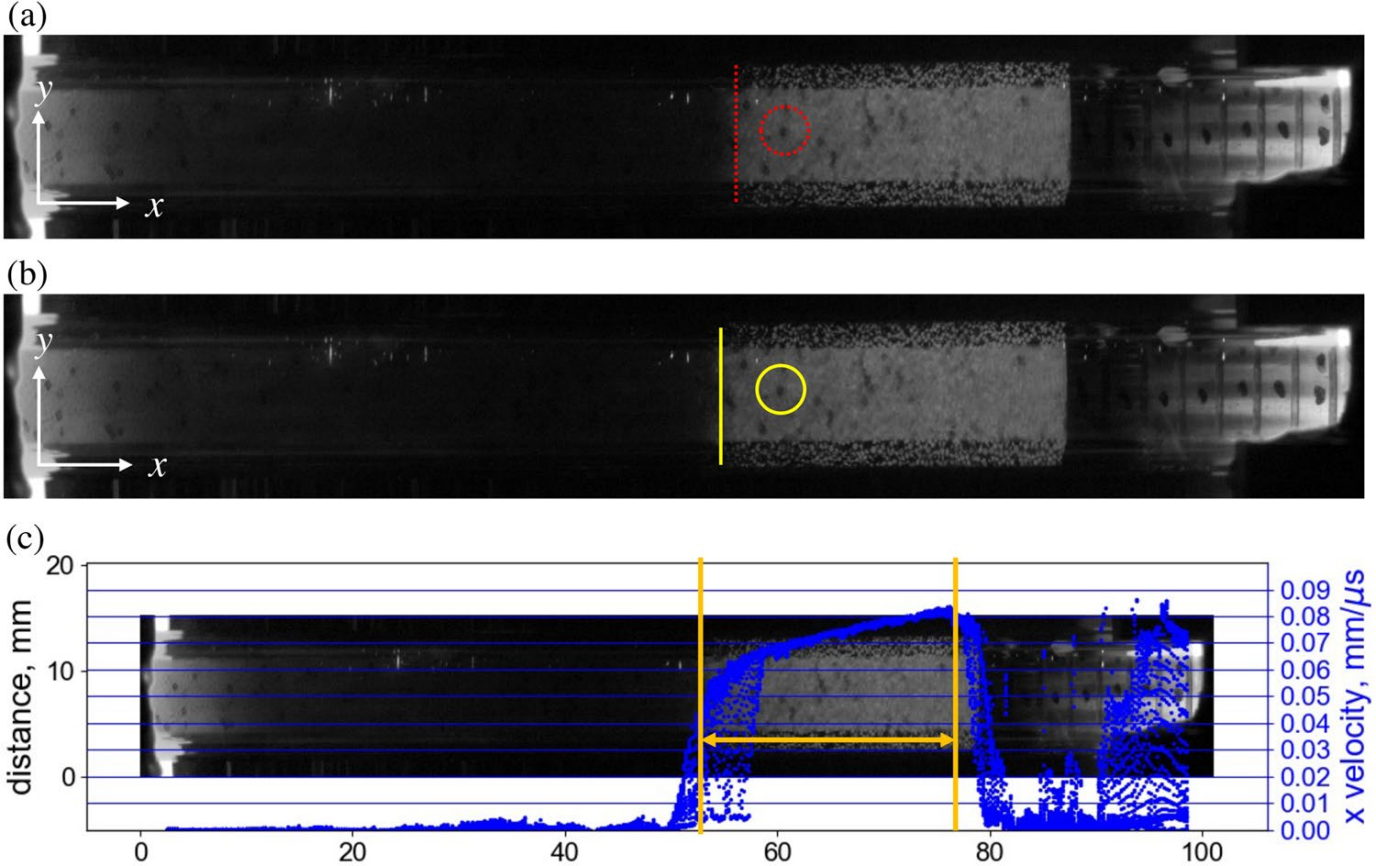
Representative high-speed image from an experiment at timesteps of (**a**) 59.5 µs and (**b**) 61.88 µs since impact. (**c**) A representative analysis from Particle Image Velocimetry (PIV) showing a distribution of particle velocities along the specimen length (x-axis indicated in Fig. 11b).

Figure 12 shows a representative high-speed image of the tested salt under shock compaction with an impact velocity of 95 m/s. The PIV method compared two successive images and tracked points on images (e.g., the point circled in Fig. 12a and b). Approximately 1,000 tracking points for PIV were utilized, and particle velocities were successfully profiled in Fig. 12c. For further analysis, considering the particle velocity in the wake of the shock wave was not uniform, we used the following analysis procedure. For the $U_s-U_p$ plot, particle velocity ($U_p$) corresponding to a timestep was obtained by averaging a spatial distribution of particle velocities between the shock compaction front and the contacting face of the piston, marked by vertical lines in Fig. 12c. The piston face was identified using the initiation of the significant negative derivative of particle velocity, while the shock compaction front was identified using the highest positive derivative of particle velocity. The shock compaction velocity ($U_s$) was calculated using the distance between the leading edges of the compaction wave in two consecutive images separated by a finite time. The leading edge is indicated using a vertical-colored line in Fig. 12a (red) and b (yellow). The leading edge is distinguished based on the contrast between pre- and post-shock compacted regions since the flood light illuminates salt crystals in densified salt regions. Specifically, the bright grey part within the sample was deemed the shock-compacted region in granular salt. The unshocked region of the salt was identified by the darker region. During the shock test, compaction velocity and particle velocity were modulated as shock compaction wave propagated through granular salt. Five compaction and particle velocity sets were calculated over the 10 mm travel distance of the shock wave from 10 mm to 60 mm of the *x*-axis along the specimen length shown in Fig. 14b. Note that for the sake of PIV and subsequent shock substructure analyses of experimental data, we used the *x*-axis indicated in Fig. 12c with origin on the left, indicated in Fig. 11b. In the case of simulations, the origin is on the right, and the *x*-axis is flipped, as indicated in Fig. 14b.

### Mesoscale modeling: particle preparation by pluviation

**Figure 13:**
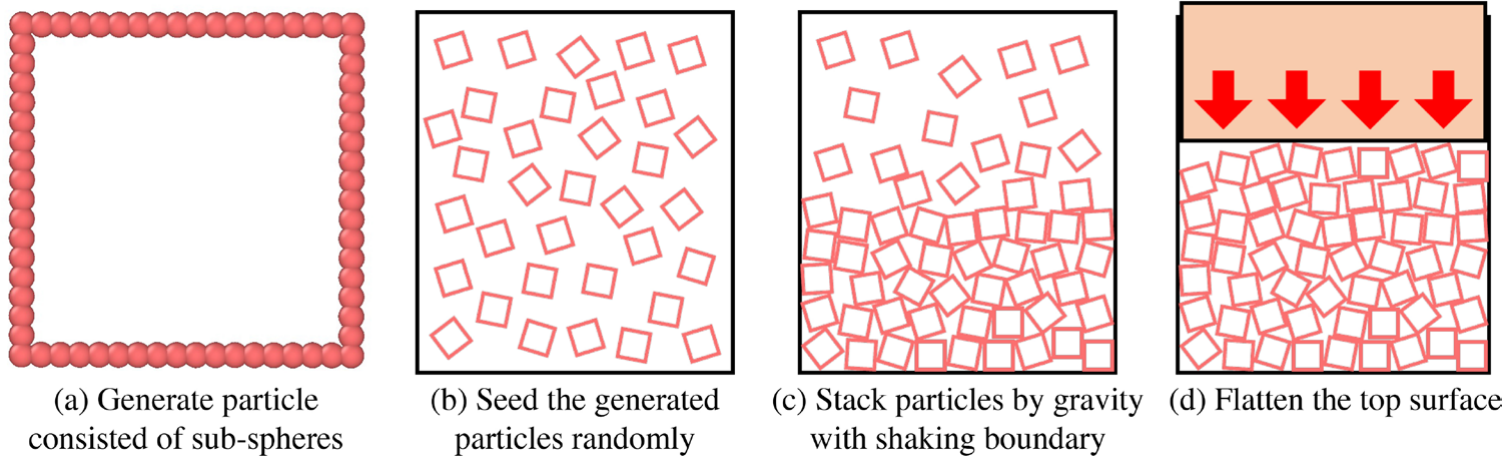
Process for dry pluviation simulation to obtain initial conditions for granular salt.

Various methods have been proposed for generating the particle domain with realistic particle arrangement, namely, the particle insertion method^41,42^, imported cross-sectional images for scanned particle sets^43,44^, and perturbation particle approach after shrinking particles^22^. An approach simulating dry pluviation in this study enabled the generation of contacting particle systems under gravity-induced body force and had to have contacting particles as an essential factor in simulating the mechanical response of interacting grains in the initial configuration.

Figure 13 illustrates the computational process for dry pluviation to generate initial conditions for granular salt used in our shock simulations. Pluviated granular sample was generated using a sequence of codes developed in Matlab and LAMMPS packages. LAMMPS (Large-scale Atomic/Molecular Massively Parallel Simulator) is a molecular dynamics package including a granular package for particle flow simulations^45^. The particle size and its distribution is controlled by Weibull distribution function with two variables in Eq. 1, where $\lambda$ is scale variable and *k* is shape variable^46^, in MatLab.

1 $$\begin{aligned} f(x;\lambda ;k)=\left\{ \begin{array}{ll} \frac{k}{\lambda }(\frac{k}{\lambda })^{(k-1)} e^{-(\frac{k}{\lambda })^k}, x\ge 0\\ 0\;\;\;\;\;\;\;\;\;\;\;\;\;\;\;\;\;\;\;\;\;\;\;, x<0 \end{array}\right. \end{aligned}$$

For mesoscale modeling, particles were generated with a mean side length of $L_{avg}=0.375$ mm using a Weibull distribution with $\lambda =3.25$ and $k=3.61$, and 2025 such particles were generated in a sample. Fig. 13a shows each square-shaped particle discretized using sub-spheres. Positions of square-shaped salt particles were randomly seeded, as shown in Fig. 13b. MatLab generates a single particle made of deformable sub-spheres with a diameter of 0.02 mm along its boundary; the sub-sphere was small enough not to significantly influence the shape of a single particle (Fig. 13). This technique is applicable for generating a random shaped particle, as well. Next, the position data of sub-spheres that form individual square-shaped salt grains were imported in LAMMPS. The discrete element method was employed for sub-spheres with the elastic constant of 30 kPa and a Hertzian interaction between particles and between a particle and a wall. Particle positions were then allowed to evolve under a gravitational accelerative force that simulated the pluviation process. Subsequently, the pluviated particles were further rearranged by applying a small sinusoidal displacement to the boundary walls simulating the tapping procedure in the experiment (Fig. 13c). After particle rearrangement, a moving wall from the top was pressed on the particles to yield a flattened top surface of the particles. Throughout this process, particle positions evolved without overlapping of sub-spheres with those belonging to other particles (Fig. 13d).

In mesoscale modeling of shock compaction, it is acceptable to consider two-dimensional simulations to avoid the expensive computational cost associated with 3D calculations^23,25^. Furthermore, comparisons between 2D and 3D simulations with spherical grains by Borg and Vogler (2013)^47^ reveal that 2D simulations with sliding (no friction) exhibited comparable trends in stress distributions, lateral velocity, and temperatures compared to 3D simulations, although, 2D simulations underestimated pressure compared to those in 3D. Our approach involves stereology analysis considering equivalent porosity in 2D corresponding to that in 3D, with the intent of obtaining consistent pressure distribution and shock compaction velocity. This technique allows our model to exhibit a 2D mesostructure similar to 3D, establishing more initial contacts between particles and better capturing the pressure and the shock compaction velocity as we anticipate in 3D. The targeted porosity for 2D configuration was calculated from porosity in 3D using the following expression (applicable to 2D and 3D):

2 $$\begin{aligned} D_X=\frac{(n_{max}-n)}{(n_{max}-n_{min} )} \end{aligned}$$

where *D* is the relative density of grain material, *X* is either 2D or 3D, and *n*, $n_{max}$ and $n_{min}$ are targeted maximum and minimum porosity, respectively. Porosity in 2D was calculated as the ratio of the pore area to the occupied container area at the end of the pressing stage following pluviation. Theoretically, $n_{(2D,max)}$ and $n_{(2D,min)}$ are 0.5 and 0, respectively, for square-shaped grains in 2D, corresponding to the chess board and structured grid patterns. $n_{(3D,max)}$ and $n_{(3D,min)}$ are 0.66 and 0 respectively for cubic grains in 3D. The porosity for 2D was obtained as $n_{2D}$= 0.27, corresponding to the porosity of the granular salt sample used in the experiments, $n_{3D}$= 0.35, based on the condition that the relative density of grain material is the same in 2D and 3D, i.e. $D_{2D}$=$D_{3D}$. If a granular sample exceeded the targeted porosity, particles were selectively removed from the pluviated sample until the targeted porosity was reached; the particles chosen for removal had the maximum coordination number amongst all particles. This process allowed the retained particles to maintain their contact with at least one other particle.

### Mesoscale model setup

FLAG is a multiphysics hydrocode developed at Los Alamos National Laboratory (LANL) and used for shock dynamics simulations. The FLAG hydrocode computes continuum mechanics solutions using finite volume formulation for fluids and solids. This study adapted FLAG hydrocode based on the Lagrangian method, embedding a computational mesh in the material domain and solving for the mesh deformation in space at discrete points in time. The domain of each grain has its boundary controlling the interaction with other grains to transmit forces and prevent interpenetration between Lagrangian grains. A mesh convergence study was performed, and an appropriate zone size was chosen (8 zones along the length of grain in this study), details of which are included in the Supplementary material. We adopted an isotropic elastic-plastic constitutive material model with linear hardening combined with a tabulated Equation of State (EOS) from Sesame tables^48^ for deformable grains. Earlier studies reported that a viscoplastic model could capture the mechanics of granular salt during creep or static loading^49,50^. An assumption of isotropic elastic-plastic hardening material is thus reasonable for a grain of salt undergoing confined compression and subsequent brittle crushing since, under confined compressive conditions, a crushed solid tends to show stress hardening rather than softening at the continuum scale^51^. Past studies on granular materials have successfully analyzed compaction using simulations without fracture and friction^22,52^. According to Borg and Vogler (2009)^23^, the dissipation due to visco-plastic work surpasses that due to grain contact and friction when material deformation is low. The simulations conducted in this study do not include a fracture model. While it is possible for the simulation in this study to include a continuum-based “Pmin” failure model^53^, similar to Borg and Vogler (2009)^23^, such an approximate approach has a tendency to introduce increased uncertainty associated with the post-failure treatment. Specifically, abruptly enforcing the shear and deviatoric stress to zero may not conserve total energy. Table 2 lists various material properties utilized for salt for mesoscale modeling.

**Table 2:** Material Properties for mesoscale simulations.

Materials	Salt^54^	Brass^55,56^	Sapphire^57,58^
Density $(\text{g/cm}^3)$	single grain: 2.16; sample: 1.41	8.47	3.97
Shear modulus (GPa)	10.48	38.7	175
Hardening modulus (GPa)	2.34	20	20
Yield strength (MPa)	11.8	337	380
SESAME table	7282	4100	7411

**Figure 14:**
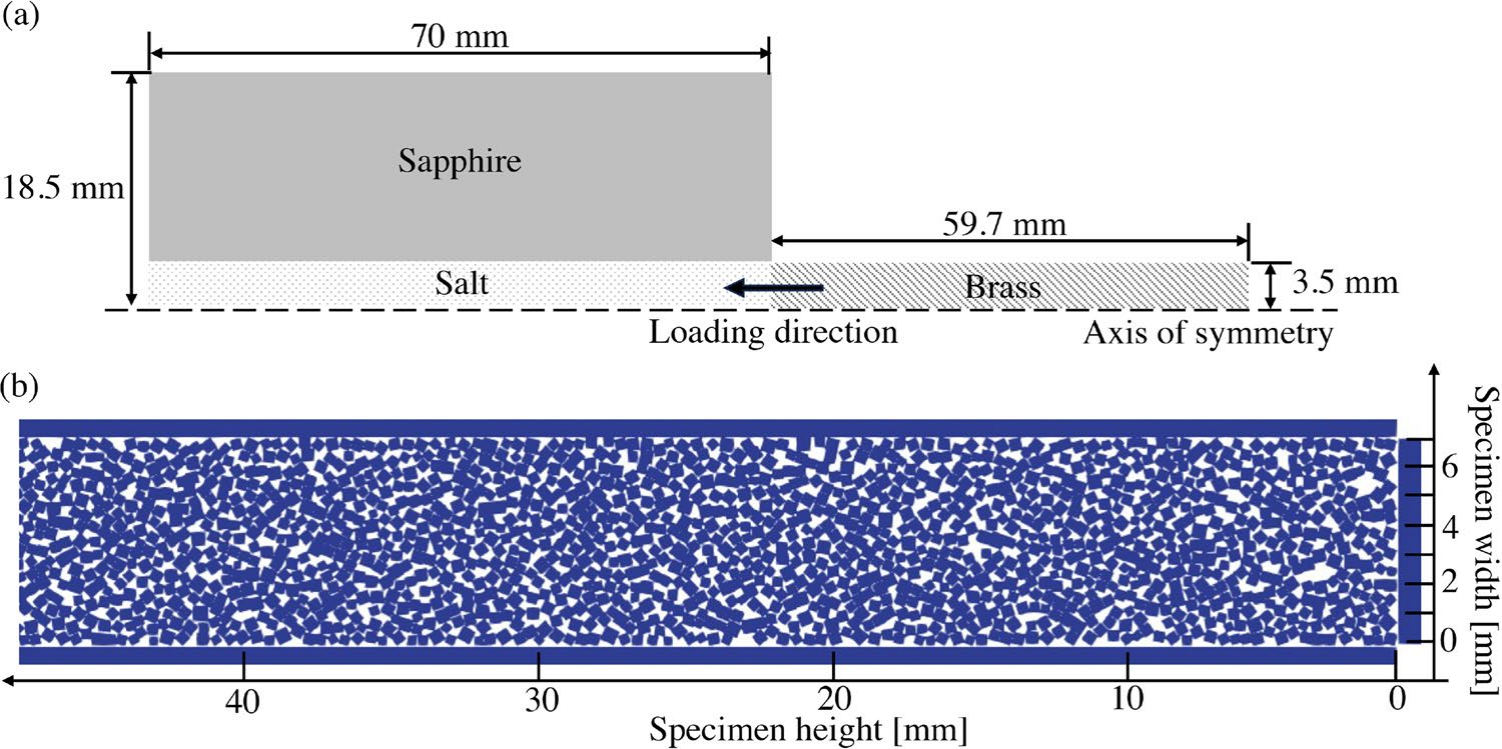
(**a**) Schematic of the linear compaction test (**b**) enlarged/cropped view of dry-pluviated granular sample.

This study hypothesizes that the mesoscale modeling excluding fracture but including plastic deformation of grains to account for dissipation would still be able to capture the response of granular salt under weak shock compaction. Table 2 summarizes the properties of materials assigned in this computational model. The material properties assigned to a salt particle were determined from the experimental measurement of rock salt (reported by Bauer et al., 2019^54^), such that the material within a single particle can be considered a continuum. Fig. 14 shows a schematic of the configuration for simulation based on the experimental setup shown in Fig. 11b. Figure 14b shows an enlarged view of a particle domain within a cylinder. Simulations were executed on high-performance computing at LANL utilizing 144 processors for about 12 hours.

### Mesoscale modeling: spatial averaging of field data

To better understand the substructure of the shock compaction wave, a 1D field pressure $\overline{p(\cdot )}$ was calculated from the 2D zone pressure. The spatial distribution of zone pressure along the *y*-axis was averaged while retaining the variation along the *x*-direction (i.e. along specimen length in Fig. 4). The spatial averaging technique utilized in this work is the Gaussian Moving Average (GMA). A Gaussian function was chosen because it can better preserve the magnitudes of the field variable gradients at the shock front compared to the uniform distribution. Unlike the simple moving window average, instead of using equal weights for each data point in the calculation, GMA uses a Gaussian weight function with the mean of the Gaussian distribution located halfway over the window length along the *x*-axis ($x_\mu$). In our analysis, the window length along the *x*-axis was 1 mm, and along the *y*-axis was the entire specimen width (bore diameter). Weights calculated in this manner were solely a function of the *x*-position of a data point relative to the mean position, i.e., $w(x')=w(x-x_\mu )$, where (*x*, *y*) are positions of zone centers, which are discrete, while $x_\mu$ can be continuous regardless of whether that location is occupied by a zone.

The Gaussian moving average can be expressed by Eq. 3, where the double integral is over the material domain within the window area.

3 $$\begin{aligned} \overline{p(x_\mu )} = \frac{\iint ~p(x_\mu +x',y)~w(x')~dx'~dy}{\iint ~w(x')~dx'~dy}~~~~\text {where}~w(x')=\frac{1}{\sqrt{2\pi \sigma }}~\text {exp}\left( {-\frac{(x')^2}{2\sigma ^2}}\right) \end{aligned}$$

The parameters that define the Gaussian distribution are standard deviation, $\sigma$, and mean, $\mu$, while *p*(*x*, *y*) is the zone pressure field, $w(x')$ is a Gaussian weighting function. The limits for integral are $x'=-0.5$ to 0.5 mm and $y=0$ to *d*, where *d* is the inner diameter of the sapphire tube. The averaging technique assumes uniform weights for zone points along the *y*-axis. Thus, this expression enables a continuum 1D analysis of the underlying discrete nature of the 2D zone pressure. Based on the gradient profile of the field pressure, we identified three regimes that make up the shock compaction wave:

(a) Precursor, $x_p$, was identified as the leading edge of the shock compaction wave, formed by elastic precursor in the material domain. The position of this front was mathematically defined using the linear gradient of the field pressure, $\Delta p$, and the precursor location was chosen for which the value of the gradient surpasses the threshold (e.g., $\Delta p$ > 1 MPa) and is given by Eq. 4.

4 $$\begin{aligned} x_p \equiv \left| \frac{dp}{dx} \right|> p_{threshold} \end{aligned}$$

(b) To identify the shock compaction front, $x_m$, the maximum first derivative value was calculated according to Eq. 5. This study defined the velocity of this compaction front as the representative shock compaction velocity for reporting subsequent modeling results.

5 $$\begin{aligned} x_m \equiv \text {max}\left| \frac{dp}{dx} \right| \end{aligned}$$

(c) Eq. 6 defines the shock compaction end, $x_c$. A uniform condition of shock pressure was reached behind this part of the substructure, known as a ‘shock state’. This position was chosen by identifying the maximum value of pressure multiplied by the second derivative of pressure, expressed as:

6 $$\begin{aligned} x_c \equiv \text {max}\left( p\left| \frac{d^2 p}{dx^2} \right| \right) \end{aligned}$$

### Supplementary Information

Supplementary Information.

## Data Availability

Data generated in this manuscript will be made available upon reasonable request and conforming to the policies of the Los Alamos National Laboratory.
