# Supplementary material for: Weak shock compaction on granular salt
Source: Sci Rep. 2024 Jul 19;14:16695. doi: 10.1038/s41598-024-67652-z (PMC11271543; doi:10.1038/s41598-024-67652-z)
Supplement: Supplementary file 1 — Supplementary Information. [file 41598_2024_67652_MOESM1_ESM.pdf]

Supplementary Material

July 2, 2024

Methods

Mesoscale Modeling

Mesh Convergence

FLAG can create a mesh of a four-node element (aka a zone) within a material domain occupied by a particle. FLAG then solves for field variables and thermodynamic quantities using material constitutive and equation of state continuum models accounting for contact between the particles. Discretizing the material domain into smaller-sized zones to obtain numerically accurate results is called mesh convergence and simultaneously assists in capturing stress gradients associated with features at lower length scales. However, using more zones for higher mesh resolution can be computationally expensive. Therefore, it is essential to determine a reasonable mesh resolution that balances the numerical solution's accuracy and efficiency. This study examined the effects of shock compaction by

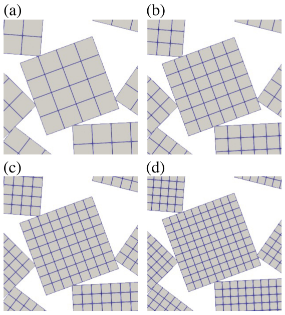

Supplementary Figure 1 – Assessment of different zone sizes along the constant particle length for establishing numerical convergence. The number of zones in a meshed particle is (a) 4 zones (b) 6 zones (c) 8 zones and (d) 10 zones.

testing four different mesh densities, namely 4, 6, 8, and 10 zones along the particle length, as shown in Supplementary Figure 1. We analyzed field-averaged pressure and velocity for a particle near the piston. Results on the field-averaged pressure for all the mesh densities, shown in Supplementary Figure 2a, are oscillating due to the phenomenon of wave propagation sub-scale to the particle and bounded by a particle boundary. A comparison of the field pressure history obtained using 4 zone mesh (red colored in Supplementary Figure 2a suggests a higher magnitude of field pressure than the result obtained using 6 zone mesh. The difference in field pressure for the 4-zone mesh was about 6.64 MPa, approximately 2.1 times higher than in other cases. Additionally, the time history of the pressure field (Supplementary Figure 2a) and the averaged particle velocity (Supplementary Figure 2b) obtained using 4 zone mesh as a function of time had a higher period of oscillation than other cases. These observations suggest a mesh resolution of 6 zones or higher would be appropriate. Supplementary Figures 2a and 2b show that the results become more consistent as mesh resolution increases from 8 zones to 10 zones. Considering the accuracy and time efficiency of obtaining numerical results, subsequent simulations used 8 zones along the length of a particle for the mesoscale simulations. Supplementary Figure 1c shows the selected case of mesh discretization.

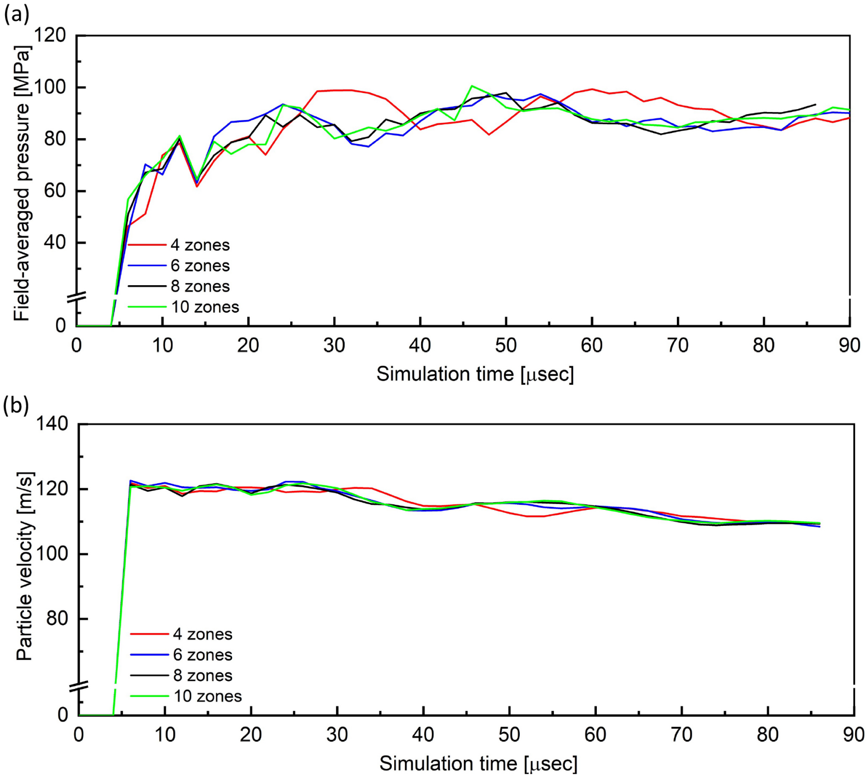

Supplementary Figure 2 – The evolution of (a) field pressure (b) particle velocity as a function of the mesh size

Shock Experiments

Visualization of the high-pressure compaction front

The mechanism that shows the arrival of the higher-pressure compaction front is known as Frustrated Total Internal Reflection. In this experiment, the illumination light was piped into the bottom of the sapphire tube. Light intersecting the tube wall at less than the critical angle ($\theta_c = 34.5\,\text{deg}$ for sapphire) will escape the tube, some of which will illuminate the salt bed while the rest will escape. Light intersecting the tube wall at the critical angle or greater will undergo total internal reflection and will continue to propagate down the tube, much like how fiber optics work. However, at the tube-air interface, some of the electromagnetic wave can propagate into the lower index of refraction material even at angles greater than the critical angle. This type of wave is known as an evanescent wave and will attenuate as it propagates into the low index of the refraction medium.

We can determine the distance at which it decays to 1/e of its original amplitude (See Introduction to Optics by Frank L. Pedrotti, S.J.; Leno S. Pedrotti; Leno M. Pedrotti) as:

$$\frac{\lambda}{2\pi\sqrt{n^2\sin^2\theta - 1}}$$

Unless the evanescent wave interacts with higher index material within roughly this distance, it will decay away. In our system, this distance is 2.5 μm at the critical angle and 50 nm at 60 deg. For much of the light, this distance is in the range of tens of nanometers, which means only the salt within this distance will reflect a significant amount of this light, especially considering the cubic form of the salt crystals. However, if the pressure of the compaction is sufficient to deform the salt crystals so that they are within this distance, much lighter will be reflected, and these salt crystals appear to 'pop' out.
